# Prediction of Thermo-Mechanical Properties of 8-Harness Satin-Woven C/C Composites by Asymptotic Homogenization

**DOI:** 10.3390/ma17061284

**Published:** 2024-03-11

**Authors:** Chenglin Ruan, Junpeng Lv, Liping Zu, Lisheng Liu, Hai Mei

**Affiliations:** 1 Department of Mechanics and Engineering Structure, Wuhan University of Technology, Wuhan 430070, China; 273077@whut.edu.cn (C.R.); parrypeng623@whut.edu.cn (J.L.); zuliping@whut.edu.cn (L.Z.); 2 Hubei Key Laboratory of Theory and Application of Advanced Mechanics, Wuhan University of Technology, Wuhan 430070, China

**Keywords:** microstructure, asymptotic homogenization, elasticity matrix, coefficient of thermal expansion, temperature dependence

## Abstract

The elasticity matrix and the coefficients of thermal expansion (CTEs) of 8-harness satin-woven (8HS) carbon-fiber-reinforced carbon matrix (C/C) composites at high temperatures were obtained by the asymptotic homogenization method (AHM) and finite element method (FEM). By analyzing the microstructure of the 8HS C/C composites, a representative volume element (RVE) model considering a braided structure was established. The effects of the temperature and component volume fraction on the elasticity matrix and CTEs of the composites were investigated. The sensitivity of model parameters, including the size of RVE model and mesh sensitivity, were studied. The optimal calculation model was employed. In addition, the effects of the 4HS methods and 8HS methods on the elastic constants of the composites were compared. The temperature and variation in the carbon fiber volume fraction were found to have a significant impact on the elasticity matrix and CTEs of composite materials. At the same volume fraction of carbon fibers, some elastic coefficients of the 4HS composite material were slightly lower than those of 8HS composite material. This research affords a computational strategy for the accurate prediction of the themo-mechanical properties of satin-woven C/C composites.

## 1. Introduction

Carbon-fiber-reinforced carbon matrix (C/C) composites exhibit a series of excellent properties such as low density, high strength, high plasticity, and impact resistance, and they are a widely used braided material [1]. In terms of the fiber reinforcement orientation, they can be divided into unidirectional (UD), two-directional, and three-directional forms. The research and application of UD fiber-reinforced composites have been quite extensive, but their transverse performance defects cannot be ignored. Nowadays, braided composites have been widely used in various fields due to their more attractive transverse characteristics than UD composites [2].

C/C composites are widely used in high-temperature environments. Their thermo-mechanical performance at high temperatures affects their application. The properties of braided C/C composites are mainly affected by the method of weaving, the material type of the fibers and matrix, and the volume fraction of each component [3]. According to different weaving methods, they can be divided into woven, knitted, and nonwoven types. In terms of weaving efficiency, manufacturing costs, and overall performance, woven fabric has more advantages than knitted fabric, and it is the most widely used fabric structure. The carbon fabric preforms of the most common 2D C/C composites are usually woven in plain, twill, and satin patterns, and the resulting C/C composites have a lower modulus than the fibers themselves due to the curvature and wavy appearance of the fiber bundles at the knitting junction.

To fully understand the role of C/C composites in high-temperature environments, their thermo–mechanical properties were investigated. Chen [4] tested the compression, bending, and tensile properties of carbon fiber/silicon (C/SiC) composites from room temperature up to 1000 °C and analyzed the fracture micromorphology of the composites. Cheng [5] first studied the bending performance of C/C composites at a temperature of 2600 °C and observed their plastic deformation at high temperatures.

Currently, tests at high temperatures (above 1600 °C) are difficult, and the high cost of material production and characterization, the time cost, and doubts about the accuracy of the results have prompted scholars to investigate the properties of composites using theoretical methods and numerical methods.

Theoretically, works based on theoretical analytical methods include those by Tran et al. [6] using the Mori–Tanaka [7] method to derive the Eshelby spherical non-uniformity problem and predict the equivalent properties of gradient composites containing spherical inclusions. The classical methods, such as the self-consistent method and Mori–Tanaka method, reflect the average of the stress and strain of representative volume elements, which can solve some problems in the calculation of the material properties, but most of them are still limited to the calculation of the linear and nonlinear basic mechanical properties of heterogeneous materials. Meanwhile, many assumptions and premises proposed by commonly used semi-analytical methods about the microstructure limit the application of theoretical methods in material research with complex microstructures. Therefore, more accurate and convenient simulation methods for composite materials have become the focus of research.

Numerically, with the wide application of large-scale commercial finite element software, RVE, a representative volumetric element method based on finite element software, has also been used to analyze the equivalent properties of composites with complex shapes. Pahr et al. [8] found that the FEM can study the macroscopic mechanical properties of heterogeneous materials in more detail than experiments. Salviato [9] analyzed the in-layer size effect of textile composite structures from both experimental and numerical aspects. Using the three-dimensional finite element method (FEM), Galatti et al. [10] performed a preliminary prediction of the mechanical behavior for composite materials fabricated by continuous filament fabrication (CFF). Srivastava [11] predicted the effect of embedding graphene sheets (GSs) into the carbon matrix of 4D-C/C composites on the elastic modulus through the FEM. The representative volume element (RVE) method has been used to predict the properties of braided composites, but it lacks a strict mathematical framework to clarify the relationship between the equivalent properties and material layout. In contrast, the asymptotic homogenization method (AHM) was born from strict mathematical theory and has a higher computational efficiency [12].

The detailed numerical calculation of the structural–mechanical behavior of composites is time-consuming, which can be significantly improved by the homogenization method [12]. The AHM is considered for the calculation of the thermodynamic properties of composite materials.

In the 1970s, scholars introduced the homogenization theory proposed by Benssousan [13] and Sanchez-Palencia [14] into the study of heterogeneous materials. This method is utilized to analyze material systems with multiple scales, enabling the connection between the microscopic scale containing the second phase space and the macroscopic scale of the overall structure. Compared to the RVE method, this approach offers advantages such as not requiring a global periodicity assumption and allowing different microstructures at various points within the macroscopic structure. Based on this method, Guedes [15] pioneered a computational program that merges the AHM with the FEM to determine the effective elastic moduli of composite materials, thereby advancing the practical application of the AHM in engineering domains. Harsanyi [16] applied the AHM to solve the equivalent properties of plate and shell structures with microstructures. Macedo et al. [17] extended this theory to the failure judgment of composites. Dutra et al. [18] integrated the AHM with commercial finite element software to compute the equivalent properties of materials, providing a comprehensive framework of the requisite procedures to further facilitate the multidisciplinary application of the AHM across various engineering fields. Wei [19] combined the AHM and the multiphase FEM to study the performance of 3D braided composites, focused on exploring the influence of voids and interphase defects on their effective thermal expansion coefficient.

In the performance prediction of woven composites, both Barile [20] and Holmes [21] conducted relevant research on plain-weave composites. Axinte [22] established six different models of satin-fabric-reinforced composites. Naylor [23] conducted tensile tests on 5-harness satin (5HS)-weave carbon fiber epoxy composites. Skinner [24] used the 5HS weaving method to enhance carbon fiber/silicon carbide matrix composite (C/SiC) CMCs and studied their nonlinear constitutive behavior considering damage. Alshahrani [25] used frame tests to investigate the deformation mechanism of 8HS carbon/epoxy prepreg under in-plane shear. Aghaei [26] experimentally compared the effects of plain, 5HS, and 8HS weaving on the mechanical properties of glass fiber/epoxy composites. The results showed a slight edge in the tensile modulus for the 8HS over the 5HS and plain weave methods.

Considering the influence of temperature on the mechanical properties of materials, Karpov [27] studied the mechanical properties of 4D C/C composites in the temperature range of 20~2800 °C and provided the reasons why the mechanical properties of the studied materials had a special temperature dependence. Priyanka [28] fabricated mixed carbon–Kevlar fiber composites using plain and 2 × 2 twill weaves and tested them for their tensile strength, compressive resistance, bending, and low-velocity impact. Cheng [29] studied the mechanical behavior of plain-weave C/C composites at high temperatures. Skinner [30] simulated the mechanical response of plain-weave (C/SiC) CMCs in the temperature range from room temperature to 1200 °C. Petkov [31] studied the weight loss and damage development of 8HS carbon-fiber-reinforced polyimide composites in high-temperature (288 °C and 320 °C) environments in air. Siddgonde [32] studied the RVE model of 5HS C/C composites and predicted their thermo-elastic properties at high temperatures but did not consider the effect of the carbon fiber volume fraction on them. Xu [33] found through experimental research that 8HS C/C composites treated at specific high temperatures exhibit better overall performance.

In this paper, considering that there is still a gap in the research on the equivalent of 8HS C/C composites related to temperature as well as the advantages of asymptotic homogenization methods in predicting the performance of periodic composite materials, we prepared 8HS C/C composites and conducted structural characterization. By combining the asymptotic homogenization method (AHM) with the finite element method (FEM), we calculated the elastic constants and thermal expansion coefficients of representative volume element (RVE) models by varying carbon fiber volume fractions at different temperatures, and we then explored the temperature dependence of the 8HS C/C composites properties. Additionally, the effects of the 4HS and 8HS weaving methods on the equivalent elastic constants of the composites were compared.

## 2. Asymptotic Homogenization Theory and Its Application

As a multi-scale analysis method, the asymptotic homogenization method [12,34] encompasses two spatial scales: micro scale and macro scale. This technique views the composite material as being composed of a repeated microstructure with a periodic and high non-uniformity.

### 2.1. Equivalent Elastic Tensor Asymptotic Homogenization Equation

Assuming that $\Omega \subset R^{3}$ is a three-dimensional simply connected bounded field with a perfectly smooth boundary, the thermo-mechanical coupling equilibrium of the structure in this region is described by the relevant dynamic equation and the heat conduction equation.

(1) $$\frac{\partial \sigma_{ij}}{\partial x_{j}}=-f_{i}$$

(2) $$\frac{\partial q_{i}}{\partial x_{i}}=0$$

On the boundary ∂Ω, the displacement $u_{i}=0$, where, $\sigma_{ij}$ represents the stress acting on Ω; $f_{i}$ is the force; $x_{j}$ is the macroscopic coordinate; and $q_{i}$ is thermal cycle vector. The stress–strain relationship in linear thermo-mechanical problems can be expressed as

(3) $$\delta_{ij}=C_{ijkl}(\varepsilon_{kl}-\varepsilon_{kl}^{T})$$

The above formula is further expressed as

(4) $$\sigma_{ij}=C_{ijkl}\varepsilon_{kl}-\beta_{ij}\Delta T$$

where $\beta_{ij}=C_{ijkl}\cdot \alpha_{kl}$; $C_{ijkl}$ is the elastic constant tensor; $\alpha_{ij}$ is the CTE; $\varepsilon_{kl}$ is the total strain; $\varepsilon_{kl}^{T}$ is thermal strain; T is the temperature field; the temperature difference is $\Delta T=T-T_{0}$; and $T_{0}$ is the initial temperature.

The expression of the thermal cycle tensor, $q_{i}$, is as follows:

(5) $$q_{i}=-\kappa_{ij}\frac{\partial T}{\partial x_{j}}$$

where $\kappa_{ij}$ is the heat conduction tensor. These coefficients follow symmetry, with $C_{ijkl}=C_{jikl}=C_{klij}$ and $\beta_{ij}=\beta_{ji}$.

The micro and macro scales are related by a very small positive number, *ε*. $y=(y_{1},y_{2},y_{3})$ is the microscale coordinate system, and $x=(x_{1},x_{2},x_{3})$ is the macroscopic coordinate system.

(6) $$\varepsilon =\frac{x_{i}}{y_{i}}$$

When $\varepsilon \to 0^{+}$, heterogeneous macrostructures can be regarded as homogeneous macrostructures.

In a two-scale system, all structural field variables, *q*, are functions of the macroscopic coordinates x and microscopic coordinates y, as $q^{\varepsilon }(x)=q(x,y)$. Its partial derivative with respect to x is expressed as:

(7) $$\frac{\partial }{\partial x_{i}}[q(x,y)]=\frac{\partial q}{\partial x_{i}}+\frac{1}{\varepsilon }\frac{\partial q}{\partial y_{i}}$$

Therefore, Equations (4) and (5) become (8) and (9), respectively.

(8) $$\sigma_{ij}(x,y)=\frac{1}{2}C_{ijkl}(y)\left\{\frac{\partial u_{k}(x,y)}{\partial x_{l}}+\frac{1}{\varepsilon }\frac{\partial u_{k}(x,y)}{\partial y_{l}}\right\}-\beta_{ij}(y)\Delta T(x,y)$$

(9) $$q_{i}(x,y)=-\kappa_{ij}(y)\frac{\partial T(x,y)}{\partial x_{j}}$$

The displacement and temperature are asymptotically expanded to an infinite series with a small parameter, *ε*, as follows:

(10) $$u_{i}(x,y)=u_{i}^{\left(0\right)}(x,y)+\varepsilon u_{i}^{\left(1\right)}(x,y)+\varepsilon^{2}u_{i}^{\left(2\right)}(x,y)+O(\varepsilon^{3})$$

(11) $$T(x,y)=T^{\left(0\right)}(x,y)+\varepsilon T^{\left(1\right)}(x,y)+\varepsilon^{2}T^{\left(2\right)}(x,y)+O(\varepsilon^{3})$$

Similarly, asymptotic expansions of stress and heat flow can be obtained, as follows:

(12) $$\sigma_{ij}^{\left(n\right)}=C_{ijkl}\left(\frac{\partial u_{k}^{\left(n\right)}}{\partial x_{l}}+\frac{\partial u_{k}^{\left(n+1\right)}}{\partial y_{l}}\right)-\beta_{ij}\Delta T^{\left(n\right)},(n=0,1,2\cdots )$$

(13) $$q_{i}^{\left(n\right)}=-\kappa_{ij}\left(\frac{\partial T^{\left(n\right)}}{\partial x_{j}}+\frac{\partial T^{\left(n+1\right)}}{\partial y_{j}}\right),(n=0,1,2\cdots )$$

After a series of formula transformations, the governing equation of the thermo-elastic problem can be expressed as [35]

(14) $$\int_{\Omega }\left\{\frac{1}{\left|Y\right|}\int_{Y}C_{ijkl}\left[\left(\frac{\partial u_{k}^{\left(0\right)}}{\partial x_{l}}+\frac{\partial u_{k}^{\left(1\right)}}{\partial y_{l}}-\alpha_{kl}\Delta T\right)\frac{\partial v}{\partial x_{j}}\right]dY\right\}d\Omega =\int_{\Omega }f_{i}vd\Omega +\int_{\partial \Omega }t_{i}vd\Gamma$$

where $C_{ijkl}$ is the elastic constant tensor; $\alpha_{kl}$ is the coefficient of thermal expansion; ΔT is the temperature difference; $f_{i}$ and $t_{i}$ are the body force and the surface force, respectively; $v_{i}$ is an arbitrary function; Y is the period of the function; and Γ is the boundary of the region Ω.

The feature functions $\chi_{i}^{kl}\left(y\right)$ and $L_{i}\left(y\right)$ can be defined to represent the relationship between *u*^(0)^ and *u*^(1)^:

(15) $$u_{i}^{\left(1\right)}(x,y)=-\chi_{i}^{kl}(y)\frac{\partial u_{k}^{\left(0\right)}(x)}{\partial x_{l}}+L_{i}(y)T(x)$$

Based on the asymptotic expansion method, the equivalent thermal performance prediction formula of composite materials considering the thermo-mechanical coupling effect is expressed as

(16) $$C_{ijkl}^{H}=\frac{1}{\left|Y\right|}\int_{Y}\left(C_{ijkl}-C_{ijmn}\frac{\partial \chi_{m}^{kl}}{\partial y_{n}}\right)dY$$

(17) $${\bar{\beta }}_{ij}=\frac{1}{\left|Y\right|}\int_{Y}\left(C_{ijkl}(y)\alpha_{kl}-C_{ijmn}(y)\frac{\partial L_{m}(y)}{\partial y_{n}}\right)dv$$

(18) $$\alpha_{kl}^{H}={\left(C_{ijkl}^{H}\right)}^{-1}\beta_{ij}^{H}$$

When the asymptotic homogenization method is used to predict the equivalent properties, the corresponding characteristic fields $\chi_{i}^{kl}\left(y\right)$ and $L_{i}\left(y\right)$ must be solved, but the complex microstructure of the composite material makes it difficult to obtain to an analytical solution of the characteristic fields. Therefore, numerical methods such as the finite element method were used to solve the characteristic fields required for the performance prediction, and the equivalent properties of the composite material were calculated based on the numerical solution of the characteristic fields.

Based on the characteristic displacement, the equivalent elastic tensor is re-expressed as follows [36]:

(19) $$C_{ijkl}^{H}=\frac{1}{\left|Y\right|}\int_{Y}C_{ijpq}(\varepsilon_{pq}^{0(kl)}-\varepsilon_{pq}^{*(kl)})dY$$

where $\varepsilon_{pq}^{0(kl)}$ is the unit strain applied to the node and $\varepsilon_{pq}^{*(kl)}$ is the characteristic strain.

According to Sigmund’s work [37], the above formula can be written in matrix form as follows:

(20) $$[C^{H}]=\frac{1}{\left|Y\right|}\int_{Y}{\left([\varepsilon^{0}]-[\varepsilon^{*}]\right)}^{T}\left[C\right]([\varepsilon^{0}]-[\varepsilon^{*}])dY$$

where *C^H^* is the equivalent elastic tensor.

Based on the finite element discrete form,  [ε]=[B][χ], $[K]=\int_{Y}{\left[B\right]}^{T}[C][B]dY$, and [f]=[K][x]; [*B*] is the derivative matrix of the form function, [K] is the stiffness matrix; and [f] is the force matrix. Then, we obtain the finite element form of the asymptotic homogenization formula for the elasticity matrix [35].

(21) $$\left[C^{H}]=\frac{1}{\left|Y\right|}{\left([\chi^{0(ij)}]-[\chi^{*(ij)}]\right)}^{T}([f^{0(kl)}]-[f^{*(kl)}]\right)$$

where [*χ*^0^] is the displacement field generated by the unit prestrain field and [χ^*^] is the characteristic displacement field obtained by solving the periodic boundary problem.

Similarly, $\;\left[K\right]\left[L\right]=[\varphi_{\alpha }]$, $[\varphi_{\alpha }]=\int_{Y}{\left[B\right]}^{T}[C][\alpha ]dY$, and $\left[\varphi_{L}]=[K][L\right]$; [*B*] is the derivative matrix of the form function; [K] is the stiffness matrix; and $\left[\varphi_{\alpha }\right]$ is the solution of the case where all the nodal degrees of freedom are constrained and the load (−1K) is applied at the nodes. Then, we obtain the finite element form of the asymptotic homogenization formula for the thermoelastic constant [35].

(22) $$\left[\beta^{H}]=\frac{1}{\left|Y\right|}{\left(\chi^{0(ij)}\right)}^{T}([\varphi_{\alpha }]-[\varphi_{L}]\right)$$

### 2.2. Periodic Boundary Condition

When applying the AHM to predict the equivalent mechanical properties of composite materials, the whole structure of the material is not required. A representative region that contains all the essential details of the microscopic structure is used. There is a boundary between the representative region and the surrounding periodic region of the composite. Therefore, when considering the boundary of the selected representative region, appropriate boundary conditions must be introduced to achieve the numerical homogenization of the effective mechanical properties of the composite. A previous study [38] has pointed out that when predicting the equivalent properties of composites, boundary conditions that satisfy Hill’s energy law [39] must be selected, including a uniform stretching boundary, a linear displacement boundary, and periodic boundary. However, the linear displacement boundary condition usually leads to higher results, while the uniform stretching boundary condition is the opposite and is a better choice than the periodic boundary condition.

For an RVE area ω, the border ∂ω is divided into two parallel parts of the relative ${\partial \omega }^{+}$ and ${\partial \omega }^{-}$, and the two parts meet as $\partial \omega ={\partial \omega }^{+}\cup {\partial \omega }^{-}$ and $0={\partial \omega }^{+}\cap {\partial \omega }^{-}$. Any material node on the surface ${\partial \omega }^{+}$ can find a corresponding point on the surface ${\partial \omega }^{-}$,and the normal vector on both surfaces satisfies $\vec{n^{+}}=-\vec{n^{-}}$. By coupling the structural field values of the corresponding nodes, the general form of the equation used is

(23) $$u_{i}^{k+}-u_{i}^{k-}=u_{i}^{RP}$$

where superscript k+ and k− represent the corresponding set of nodes on two parallel surfaces of the cell, and $u_{i}^{RP}$ is the perturbation applied to the reference point. At the same time, it is necessary to classify the nodes on the boundary so that the boundary node traversal is not repeated.

### 2.3. Finite Element Implementation of AHM

(1) Calculate the elasticity matrix.

Step 1: Construct and apply a six-node displacement field $\left[\chi^{0\left(kl\right)}\right]$ on the RVE and solve to obtain the node reaction force $\left[f^{0\left(kl\right)}\right]$.

Step 2: Apply the node reaction field $\left[f^{0\left(kl\right)}\right]$ and the periodic boundary conditions (Equation (23)) on the RVE. Apply a fixed constraint to one vertex of the model. Then, solve to obtain the characteristic displacement field $\left[\chi^{*\left(kl\right)}\right]$.

Step 3: Apply the characteristic displacement field $\left[\chi^{*\left(kl\right)}\right]$ on the RVE and solve to obtain the nodal reaction force $\left[f^{*\left(kl\right)}\right]$.

Step 4: Calculate the elastic constants, $C^{H}$, from Equation (21).

(2) Calculate the CTE.

Step 1: Apply thermal loads (−1K) on each node with the boundary condition that all the nodal degrees of freedom are constrained and solve to obtain the corresponding nodal reaction force $\left[\varphi_{\alpha }\right]$.

Step 2: Apply the nodal reaction force $\left[\varphi_{\alpha }\right]$ and periodic boundary conditions (Equation (23)) on the RVE and solve to obtain the characteristic displacement field [L].

Step 3: Apply the characteristic displacement field [L] on each node and solve to obtain the corresponding nodal reaction force $\left[\varphi_{L}\right]$.

Step 4: Calculate the effective thermoelastic constant, $\beta^{H}$, from Equation (22).

Step 5: Calculate the effective CTE from Equation (18).

### 2.4. Verification of Asymptotic Homogenization

In order to explore the correctness of the calculation model, the same model was used in this section, and the size of RVE and grid were adjusted according to the size given in the literature.

Since it is not common to study the elastic properties of 8HS C/C composites with detailed model data, the correctness of the calculation model was explored using a 4HS C/C composite instead. The model building and property prediction methods used in this study were adopted to predict the elastic properties of 4-harness satin-weave-reinforced composites (4HS), and a single-cell model was established according to the sizes in the literature [40]. The adopted carbon fiber was T800-12K. The matrix was a shape-memory resin. The volume fractions of the fiber and resin were 45% and 55%, respectively.

The calculation results of this article and the experimental results of the reference [40] are shown in Table 1. The results in the reference study were obtained by experiments, and the experimental results were affected by the test method and material defects. The calculation in this paper did not take into account other adverse factors affecting the performance. The calculated performance was ideal, the overall value was close, and the calculation error was less than 6%, so it was considered that the calculation accuracy of this method was reasonable.

## 3. Preparation and Characterization of 8HS C/C Composites

### 3.1. Preparation of 8HS C/C Composites

**Figure 1:**
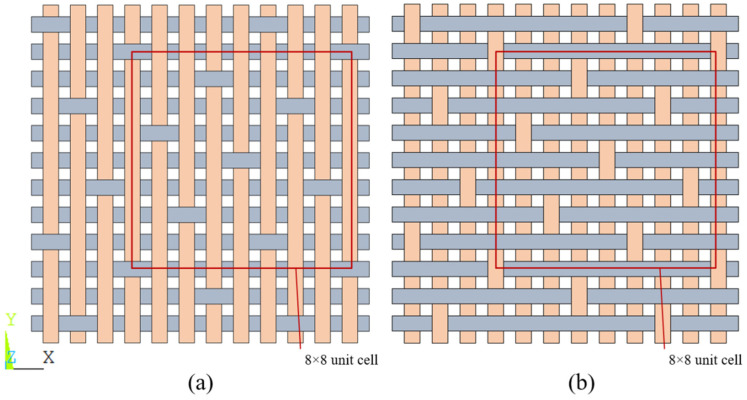
Schematic diagram of 8HS weave structures. (**a**) Plane structure of longitude, (**b**) plane structure of Latitude.

T700-12K carbon fiber was used as the raw material to prepare 8HS (shown in Figure 1) prefabricated carbon cloth. After impregnating the prefabricated resin, the material was densified to around $1.7\;g/{\mathrm{cm}}^{3}$ using alternating graphitization and chemical vapor deposition (CVD) technology to obtain a two-dimensional C/C composite material.

### 3.2. Observation of the Structure of 8HS C/C Composites

To enhance the accuracy of predicting the elastic coefficients of the composites, it was imperative to thoroughly characterize both the composite sample and its microstructure dimensions, ensuring that the established RVE model could precisely forecast material properties.

**Figure 2:**
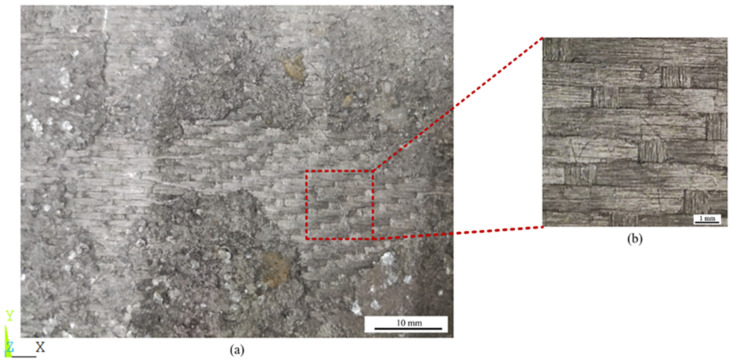
XY plane morphology of prepared 8HS C/C composites. (**a**) Actual material in the XY plane, (**b**) the minimum period in the XY plane.

Figure 2a shows the prepared 8HS C/C composites. Due to the processing technology, the material surface was enriched with deposited graphite and visible crack defects. The structural details of the XY plane are shown in the Figure 2b. In both the X and Y directions, weaving was performed every seven fibers, and the relative positions of the weaving nodes were regularly staggered. The XY plane’s weaving structure had an obvious periodicity, as shown in Figure 2.

**Figure 3:**
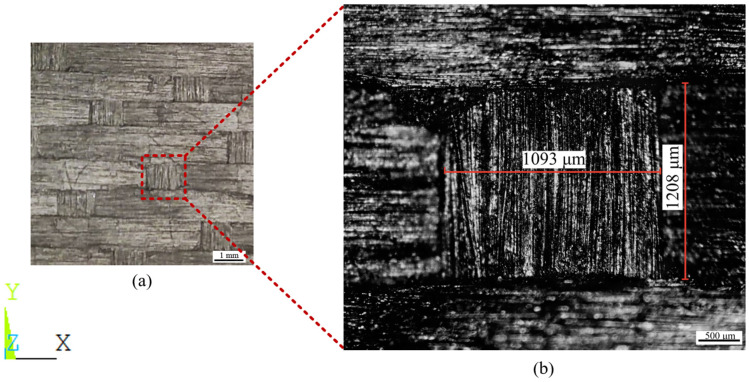
Dimensions of fiber interweaving points observed by optical microscope (OM). (**a**) The minimum period in the XY plane, (**b**) fiber intersection point.

To characterize its microstructure, we cut a small piece of sample from the whole sample and observed its structure and related dimensions by combining the use of both a camera and a metallographic optical microscope. As shown in Figure 3, the dimensions of longitudinal fibers and latitudinal fibers are characterized by examining magnified interweaving points of the fibers.

As shown in Figure 3, we randomly selected and measured the fiber width in the X and Y directions and the distance between two adjacent fibers in ten groups. The fiber width and axial spacing were obtained.

**Figure 4:**
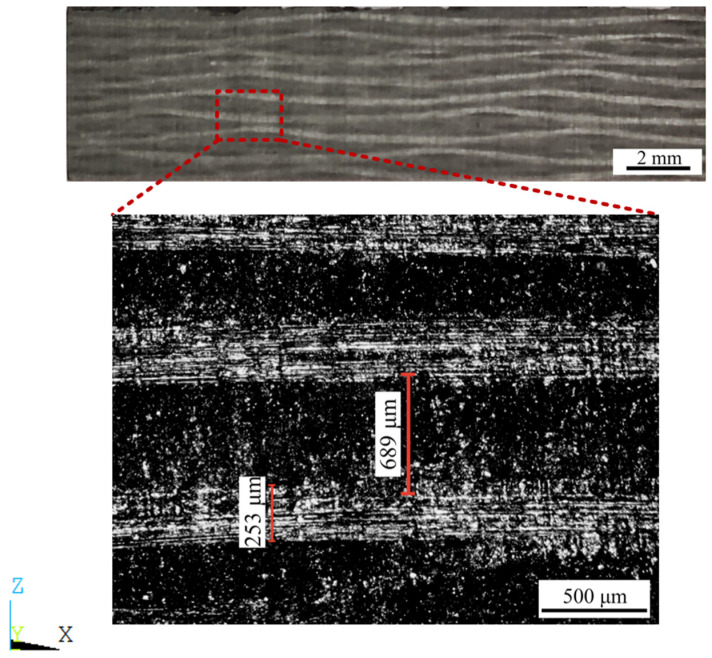
Observation map of sample thickness (Z) direction observed by OM.

**Figure 5:**
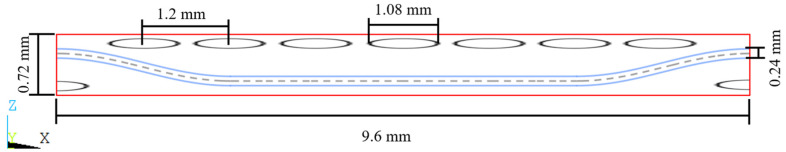
Schematic diagram of model size structure. Where, the red rectangle represents the composites unit cell, the blue curve represents the longitudinal section of the X-direction fiber bundle, and the black ellipse represents the transverse section of the Y-direction fiber bundle.

Figure 4 is a local observation map of the thickness direction (Z). We obtained the thickness of the fiber and the thickness of the single-ply structure by the same method, and we took a weighted average of the data. Detailed data are shown in below (Table 2 and Figure 5).

### 3.3. Curve Shape and Cross Section Equation of Fiber Bundle

**Figure 6:**
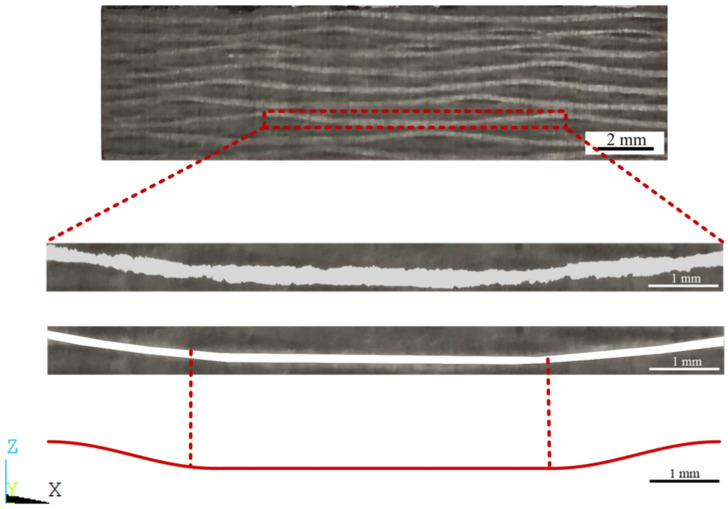
Simplification of fiber bundle and curve fitting.

To determine the detailed fiber curve equation for the subsequent finite element model, a periodic image of a single fiber was extracted from the local observation map in the thickness direction (Z), as shown in Figure 6, and the edges of the fiber part of the image were smoothed before fitting the curve.

**Figure 7:**
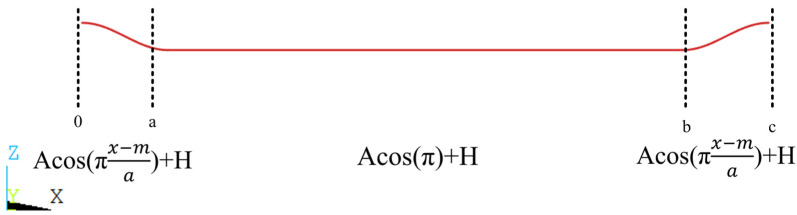
The fitting equation of fiber curve. Where, a and b are the fitting function segmentation points, and c represents the fiber length of one period.

Considering the impact of the complexity of the fitted polynomial form on the subsequent modeling, we found that the middle part of the fiber was approximately straight due to the vertical fiber compression above and the pulling effect at both ends of the fiber itself, while the two ends of the fiber were raised due to the interlacing with the vertical fibers. The fiber linear equation based on the observation results was simplified, the middle part was treated as a straight line, the fiber junction points were fit using the cos function, and the middle part was taken as the endpoint tangent of the cos function, as shown in Figure 7.

For the cross-sectional shape of the fibers, based on experimental observations and relevant descriptions in the literature [41,42], we assumed that the fiber cross-section was elliptical. Combining the observed fiber width and thickness data, we took the fiber width as the major axis of the ellipse and the fiber thickness as the minor axis of the ellipse.

Based on the node coordinates (x, y, z) and the fiber equation (see Figure 7), we obtained the midpoint coordinates (y_0_, z_0_) of the fiber cross-section (elliptical surface) where the node was located. Then, we input y, z, y_0_, z_0_ into Equation (24) to determine the element properties that the node was in contact with.

Taking the X-direction fiber as an example, its transverse cross section was parallel to the YZ plane, and the equation of cross-section can be expressed as

(24) $$\frac{y^{2}}{{\left(\frac{a}{2}\right)}^{2}}+\frac{z^{2}}{{\left(\frac{b}{2}\right)}^{2}}\leq 1$$

## 4. Construction of RVE Model

The representative volume element requires the model size to be the minimum size that includes the microstructural features. The size of the RVE must be small in relation to the overall size and should contain macroscopically uniform typical structures. A reasonable RVE model is of great significance to saving computing resources and reducing computing time.

### 4.1. Modelling Strategy

According to the characterization results, the corresponding modeling program was proposed.

**Figure 8:**
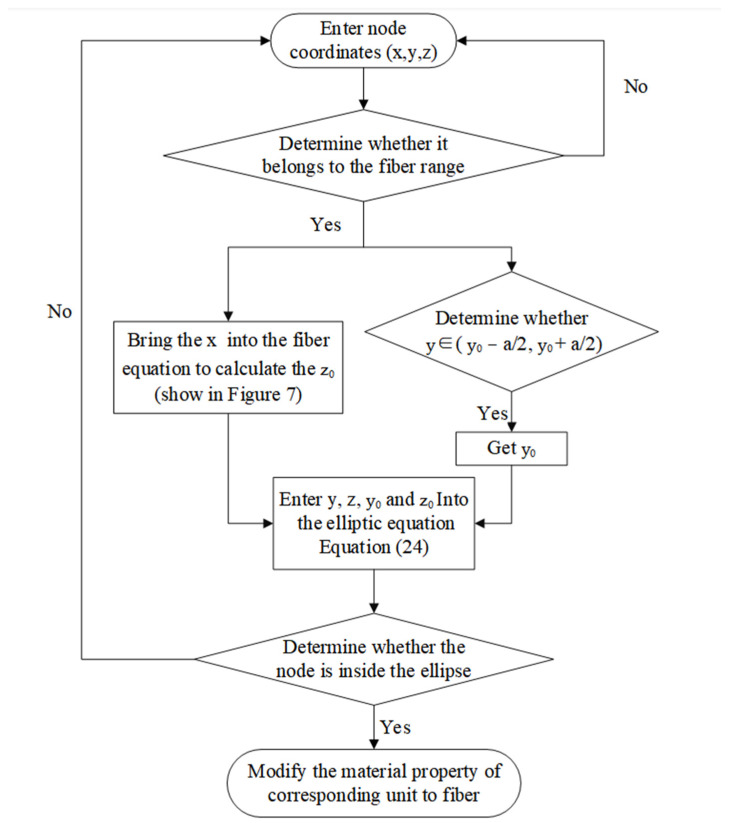
Flowchart of step 2 (the fiber generation program) in modeling.

The construction of RVE model was divided into two steps:

Step 1: The matrix finite element model conforming to the size conditions of RVE was established to obtain the relevant information of the model;

Step 2: The model nodes and units were distinguished, and the type of each component unit was determined (see Figure 8).

**Figure 9:**
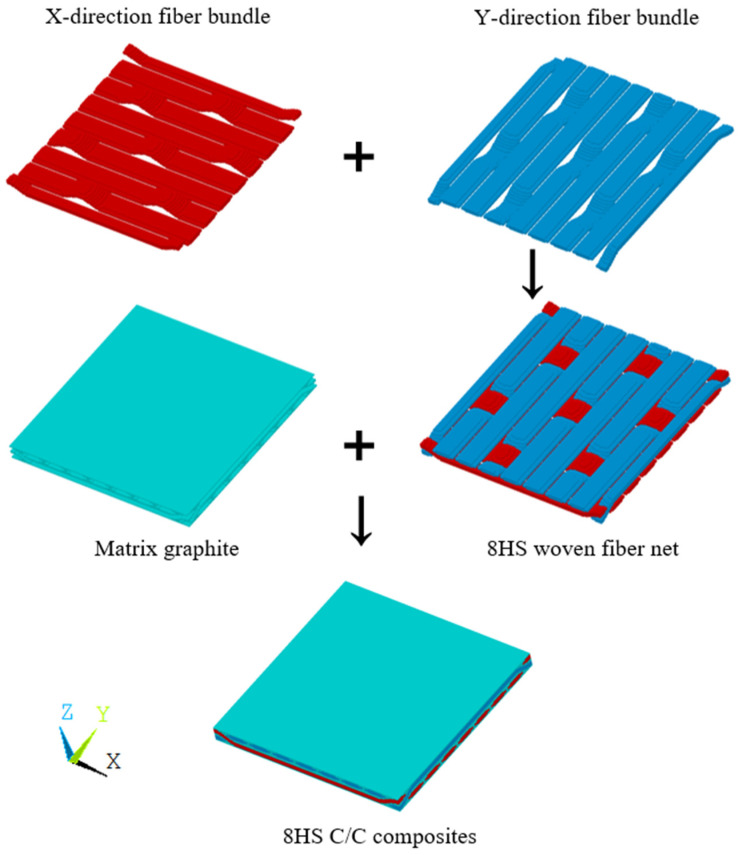
The RVE of 8HS C/C composites.

According to steps shown in Figure 9, the structural distribution of each component necessary for the establishment of the finite element model can be obtained. In order to generate the finite element model of the composite RVE, a regular hexahedron composed of the matrix material was first generated in the program, and the hexahedron grid was divided into a discrete finite element model according to the discrete number S. Finally, the matrix finite element model was judged by the linear equation of the fiber bundle and the control coefficient of the fiber cross-section, and the final RVE model was obtained.

### 4.2. Component Material Parameters

The RVE model consisted of two parts: the fiber bundle and the matrix. The fiber bundle was composed of two phases of carbon fiber and the matrix, which can be considered as a linear elastic transversely isotropic material. The carbon matrix was an isotropic material. The elastic constant of the fiber bundle was calculated by the following micromechanical formula [43]:

(25) $$E_{11}=V_{cf}E_{cf11}+(1-V_{cf})E_{m}\;E_{22}=E_{33}=\frac{E_{m}}{1-\sqrt{V_{cf}}(1-E_{m}/E_{cf22})}\;G_{12}=G_{13}=\frac{G_{m}}{1-\sqrt{V_{cf}}(1-G_{m}/G_{cf12})}\;G_{23}=\frac{G_{m}}{1-\sqrt{V_{cf}}(1-G_{m}/G_{cf23})}\;\upsilon_{12}=\upsilon_{13}=V_{cf}\upsilon_{cf12}+(1-V_{cf})\upsilon_{m}\;\upsilon_{23}=\frac{E_{22}}{2G_{23}}-1$$

where $\;V_{cf}$ is the volume fraction of carbon fiber, and $\;V_{cf}$ = 0.742 was taken from Ref. [44]; subscript  cf is the correlation properties of the carbon fiber, and subscript m is the correlation properties of the carbon matrix. *E*_11_ and *E*_22_ are the elastic modulus of the carbon fiber bundle in the longitudinal and transverse directions, respectively, and *G*_12_ and *G*_23_ are the shear modulus of the carbon fiber in the 1–2 and 2–3 planes, respectively. *v*_12_ is Poisson’s ratio of the carbon fiber bundle.

The mechanical properties of the carbon fiber bundles were calculated by substituting the data from Table 3 and Table 4 into Equation (25). These material parameters of the carbon fiber (Table 3), the carbon matrix (Table 4), and the carbon fiber bundle (Table 5 and Table 6) are listed in Table 3, Table 4, Table 5 and Table 6, respectively.

Here, unless otherwise stated, the relevant material performance parameters of each component used in all calculations were as follows:

**Table 3:** Mechanical properties of carbon fiber [44].

*T* (K)	*E*_11_ (GPa)	*E*_22_ (GPa) = *E*_33_ (GPa)	*G*_12_ (GPa) = *G*_13_ (GPa)	*G*_23_ (GPa)	*v*_12_ = *v*_13_
300	233.13	23.11	8.97	8.23	0.200
500	232.82	23.08	8.96	8.22	0.200
700	231.79	22.98	8.92	8.18	0.200
900	231.17	22.92	8.89	8.16	0.200
1100	230.78	22.88	8.88	8.15	0.200
1300	229.36	22.74	8.83	8.10	0.200
1500	227.15	22.52	8.74	8.02	0.200
1700	221.81	21.99	8.53	7.83	0.200
1900	210.63	20.88	8.10	7.44	0.200
2100	186.41	18.48	7.17	6.58	0.200
2300	157.30	15.59	6.05	5.55	0.200

**Table 4:** Carbon matrix properties at high temperature [44,45].

*T* (K)	*E* (GPa)	*v*	*α* (10^−6^/K)	*C*_P_ (J/kg/K)	*k* (W/m/K)
300	11	0.2	1.20	726.4	150.5
500	11.08	0.2	1.45	1200.5	118.5
700	11.3	0.2	1.85	1532.0	88.4
900	11.55	0.2	2.15	1703.5	73.6
1100	11.85	0.2	2.36	1820.1	63.9
1300	12.42	0.2	2.54	1939.4	56.7
1500	13.54	0.2	2.7	2003.5	51.2
1700	15.01	0.2	2.86	2040.2	47.8
1900	16.19	0.2	3.02	2075.8	45.4
2100	17.03	0.2	3.18	2106.0	43.6
2300	16.97	0.2	3.34	2138.6	42.9

**Table 5:** Calculated mechanical properties of carbon fiber bundle.

*T* (K)	*E*_11_ (GPa)	*E*_22_ = *E*_33_ (GPa)	*G*_12_ = *G*_13_ (GPa)	*G*_23_ (GPa)	*v*_12_ = *v*_13_	*v*_23_
300	175.82	20.05	8.54	7.96	0.20	0.26
500	175.61	20.07	8.55	7.96	0.20	0.26
700	174.90	20.10	8.55	7.95	0.20	0.26
900	174.51	20.17	8.55	7.96	0.20	0.27
1100	174.30	20.27	8.58	7.99	0.20	0.27
1300	173.39	20.39	8.61	8.00	0.20	0.27
1500	172.04	20.62	8.65	8.03	0.20	0.28
1700	168.46	20.66	8.59	7.97	0.20	0.30
1900	160.46	20.07	8.29	7.69	0.20	0.31
2100	142.71	18.26	7.48	6.92	0.20	0.32
2300	121.09	15.77	6.41	5.92	0.20	0.33

**Table 6:** Thermal properties of carbon fiber bundle [44].

*T* (K)	*α*_11_ (10^−6^/K)	*α*_22_ (10^−6^/K)	*C*_P_ (J/kg/K)	*k*_11_ (W/m/K)	*k*_22_ (W/m/K)
300	−0.69	5.38	1673.95	186.85	192.01
500	0.38	6.24	1868.90	173.54	179.92
700	0.98	6.45	2032.00	160.77	165.98
900	1.44	6.58	2134.36	152.60	155.76
1100	1.78	6.54	2199.52	147.55	148.47
1300	2.02	6.50	2237.26	139.71	139.05
1500	2.24	6.49	2261.47	132.08	130.35
1700	2.42	6.51	2258.19	126.86	124.48
1900	2.59	6.51	2251.48	122.95	120.12
2100	2.68	6.52	2239.38	119.26	116.29
2300	2.67	6.49	2221.45	115.84	113.23

### 4.3. Determination of RVE Parameters

To construct an RVE model, the model size is the minimum size that includes microstructural characteristics. In order to predict the material properties accurately and save on computational costs, it is very necessary to adopt the appropriate RVE model parameters. The number of equidistant scattered points, *S*, determines the mesh size of the RVE and affect the accuracy of the model calculation. In this section, the rationality of the size of the RVE model and the influence of S on the prediction results are discussed through the quantitative prediction of the mechanical properties, and the subsequent calculation of the RVE model is determined.

#### 4.3.1. Size Sensitivity Analysis

The size of the RVE model affects the calculation accuracy, and it is necessary to explore the impact of the RVE size on the simulation results and its impact patterns.

**Figure 10:**
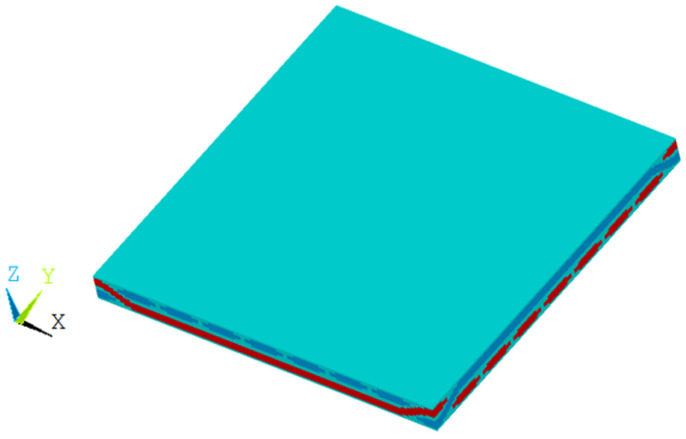
Basic computational RVE model.

The model shown in Figure 10 is the basic model, in which the reinforcement fiber and graphite matrix account for 50% each. In order to investigate the influence of different RVE sizes on the calculation results, the basic model was extended according to the fiber tiling direction and the vertical direction, respectively, and the RVE models with different sizes were generated.

**Figure 11:**
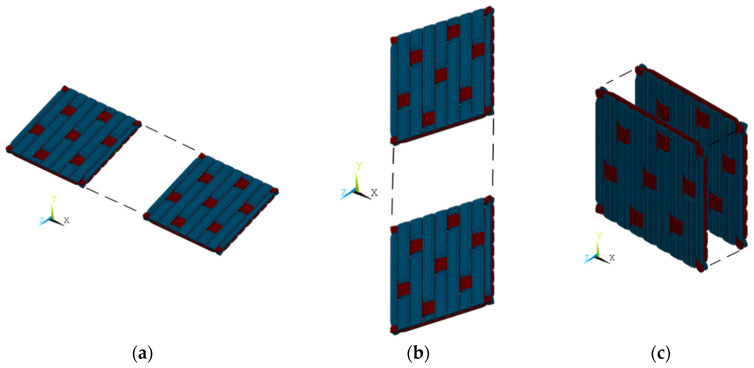
RVE extended graph (without matrix phase). (**a**) X direction. (**b**) Y direction. (**c**) Z direction.

In order to study the influence of the RVE size, RVEs with different sizes were constructed by increasing *x* and *y* times in the fiber lay-up (X and Y direction) and *z* times in the vertical direction (Z direction) to study the sensitivity of the model parameters (Figure 11).

According to composite theory, 8HS C/Cs are orthogonal anisotropic materials, and the elasticity coefficient matrix of these materials includes nine independent parameters. The elastic constants matrix and the CTE matrix are given below.

(26) $$C^{H}=\left[\begin{array}{cccccc} C_{11} & C_{12} & C_{13} & 0 & 0 & 0\\ & C_{22} & C_{23} & 0 & 0 & 0\\ & & C_{33} & 0 & 0 & 0\\ & & & C_{44} & 0 & 0\\ & & & & C_{55} & 0\\ & & & & & C_{66} \end{array}\right]$$

(27) $$\alpha =\left[\begin{array}{ccc} \alpha_{11} & & \\ & \alpha_{22} & \\ & & \alpha_{33} \end{array}\right]$$

Below are the results of the calculation for a temperature of 300 K and a number of discrete points, S, of 160.

(28) $$C^{H}=\left[\begin{array}{cccccc} 49.481 & 4.172 & 4.062 & 0 & 0 & 0\\ & 49.479 & 4.061 & 0 & 0 & 0\\ & & 15.767 & 0 & 0 & 0\\ & & & 5.984 & 0 & 0\\ & & & & 5.984 & 0\\ & & & & & 6.449 \end{array}\right]$$

(29) $$\alpha =\left[\begin{array}{ccc} 0.288 & & \\ & 0.287 & \\ & & 4.177 \end{array}\right]$$

From Equation (26), it can be seen that $C_{11}\approx C_{22},C_{13}\approx C_{23},C_{44}\approx C_{55}$, and $\alpha_{11}\approx \alpha_{22}$. The analysis suggests that although there were no isotropic planes in the three orthogonal elastic performance symmetry planes of the 8HS C/C composites, the fiber bundles in the X and Y directions (1 and 2 directions, respectively) were woven in the same way. Meanwhile, considering that the performance of woven composites is mainly influenced by the fiber volume fraction and the weaving method in the structure [1], the same weaving method and carbon fiber volume fraction resulted in highly similar cross-sectional structures and similar performance values in the X and Y directions [32]. To avoid repetition, the subsequent computational analyses will only show the effect of each factor on the elastic constants $C_{11},C_{12},C_{13},C_{33},C_{44},C_{66}$ and the CTE $\alpha_{11}$, $\alpha_{33}$.

Table 7 shows the calculation results of the RVE extended model shown in Figure 11. Although the variation in RVE dimensions in all three directions had an impact on the calculated results, compared with the mean value, the largest difference in the elastic tensor components appeared in the *C*_33_ of model XI, with a maximum value of 0.026 GPa and a relative error of 0.163%. The maximum difference between the elastic tensor of model I and model XI was 0.062 GPa. Through the comparison, it can be seen that whether increasing the number of basic units along X, Y, or Z direction or increasing the number of the basic unit in three directions at the same time, the effect on the elastic tensor was small, and the calculation results of the basic units were reliable. Considering both computational accuracy and cost, the size of the basic model (shown in Figure 10) was determined as the RVE size for the subsequent calculations.

#### 4.3.2. Finite Element Mesh Sensitivity Analysis

**Figure 12:**
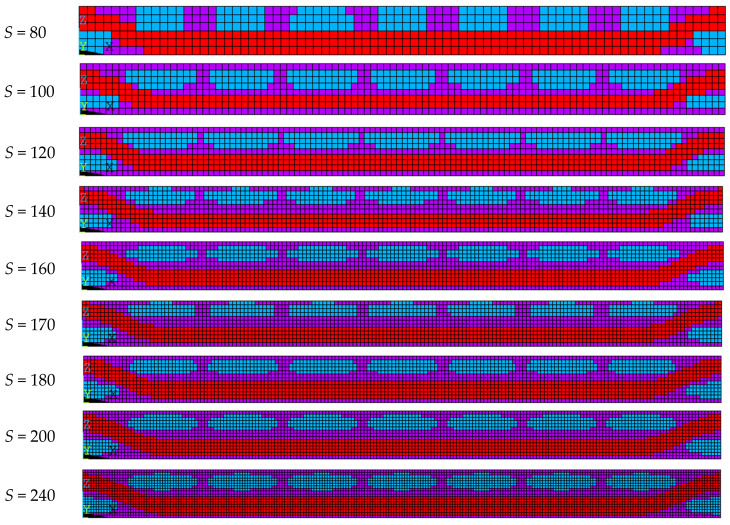
The construction of different equidistant scattered points *S*. Where, purple represents the graphite matrix, red represents the X-direction fiber bundle, and blue represents the Y-direction fiber bundle.

To explore the influence of the number of equidistant scattered points *S* (Figure 12) on the prediction of the material properties, models with the same material components but different element numbers were established to study the sensitivity of the mesh size. The dimensions in the X and Y directions of all the models were 9.6 mm and 9.6 mm, but the dimensions in the Z direction were affected by the mesh size. The elliptic fiber had a major axis of 1.08 mm and a minor axis of 0.24 mm. Theoretically, the volume fraction of the fiber in the X and Y directions was 25%. The influence of *S* on the volume fraction of each component in the models and the mesh number of models are shown in Table 8.

**Figure 13:**
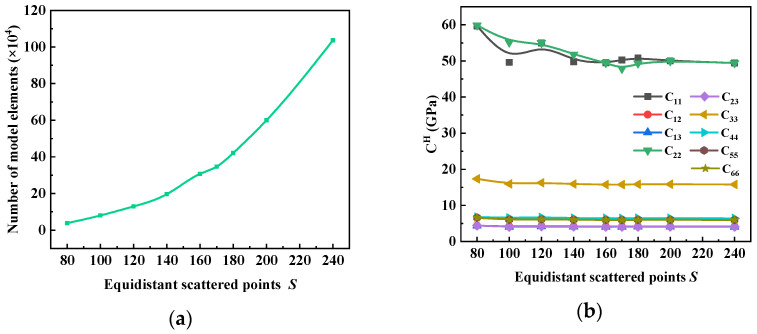
The influence of the number of equidistant scattered points, *S*, on the (**a**) model size and (**b**) elastic coefficients.

It can be seen from Figure 13 that with the increase in the number of equidistant scattered points, *S*, the number of elements in the RVE model increased nearly exponentially. In addition, the number of equidistant scattered points *S* had an effect on the volume proportion of each component of the material, resulting in fluctuations in the performance prediction results. After S=160, the volume proportion of each component tended to be stable, and the performance prediction results converged. Considering the computational costs, model I with S=160 was selected as the subsequent performance prediction RVE model.

## 5. Results and Discussion

In this section, the effects of the temperature and fiber volume fraction on the elastic properties of the materials are discussed. In order to ensure the partial change in the fiber shape and eliminate the influence of the fiber structure on the performance prediction, the RVE model with different fiber volume fractions was established by adding a single layer of the matrix. In addition, the effects of the 4HS and 8HS braiding methods on the material properties are also discussed.

### 5.1. Effect of Temperature on Thermo-Mechanical Properties of Composites

#### 5.1.1. Elasticity Coefficient

We calculated the material properties by taking a temperature point with a spacing of 200 K within the temperature range from room temperature (300 K) to ultra-high temperature (2300 K) to calculate the material properties, and we discuss the effect of the temperature on the material properties based on the results. The following is the effect of the temperature on models containing different volume fractions of carbon fibers.

Within the temperature range from 300 K to 2300 K, the elastic matrix components of the composite materials with different carbon fiber contents showed an increasing trend followed by decreasing trend with temperature.

**Figure 14:**
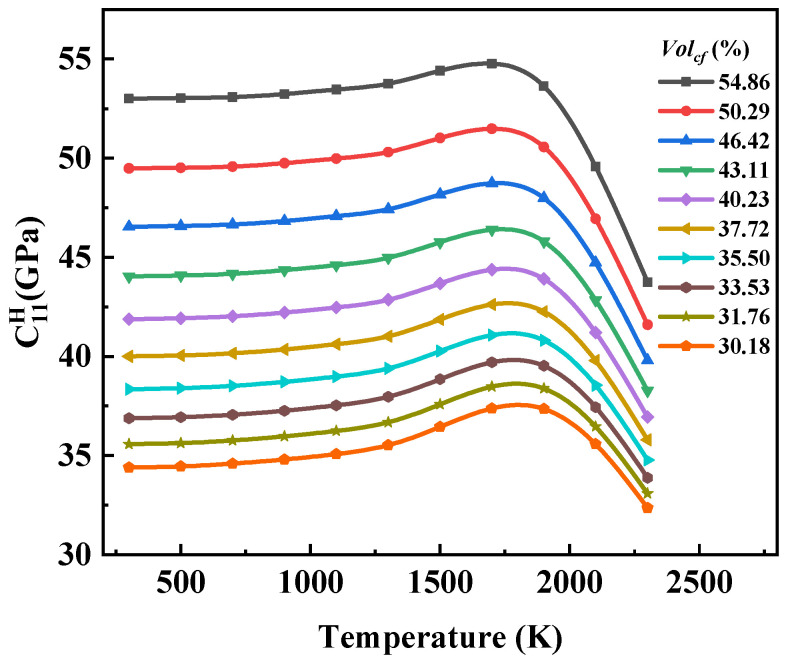
The influence curve of temperature change on $C_{11}^{H}$.

As shown in Figure 14, for the models with different volume fractions of carbon fiber ($Vol_{cf}$), $C_{11}^{H}$ stayed in the range of 300–1000 K and increased significantly in the range of 1000–1700 K. After that, its value decreased significantly with the increase in temperature, and it was ultimately lower than its room-temperature performance. In addition, at the same temperature, the performance of high $Vol_{cf}$ was higher than that of low $Vol_{cf}$, and the curve trend was more consistent with the carbon fiber performance curve. This is because the fiber was the main bearer of the composite material under tensile conditions, and the matrix mainly played the role of transferring the load. In addition, since the carbon fiber bundle is a transversely isotropic material, the fiber bundle perpendicular to the direction of load mainly participated in the load bearing with the transverse performance. The axial properties of the composites were mainly dependent on the properties of the carbon fiber bundles and affected by the matrix. In addition, an increase in the proportion of matrix phase increased the influence of the matrix on the composite materials. Therefore, compared to the sharp decline in performance of the composite materials with $Vol_{cf}=54.86\%$ after 1700 K, the rate of declining for $Vol_{cf}=30.18\%$ slowed down.

**Figure 15:**
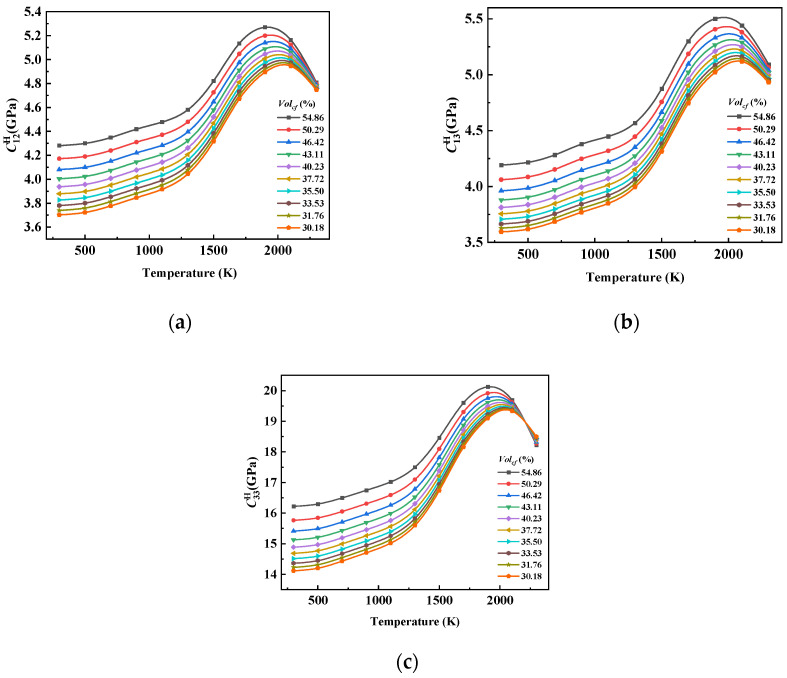
The influence curve of temperature change on (**a**) $C_{12}^{H}$, (**b**) $C_{13}^{H}$, and (**c**) $C_{33}^{H}$.

As shown in Figure 15, the variation law of $C_{12}^{H}$, $C_{13}^{H}$, and $C_{33}^{H}$ with temperature was similar, there was no significant change within 500 K, the performance increased gently in the temperature range of 500–1300 K, the performance increased sharply in the temperature range of 1300–1900 K, and it reached the maximum value around 1900 K, after which the performance declined. $C_{33}^{H}$ is numerically different from $C_{12}^{H}$ and $C_{13}^{H}$, and the curve trend of the three with temperature changes was similar to graphite. In addition, the decline rate of the three after 1900 K changed to become affected by the graphite properties and fiber volume fraction, and the degree of the influence was different. It can be seen from the curve intersection position that $C_{33}^{H}$ was more affected by the graphite properties. This is because from the perspective of the Z direction, the connection between the reinforced fiber and the graphite matrix in the composite material was similar to a series structure. According to the theory of micromechanics [46], if the bulk fraction of a reinforced fiber is not large enough, it will not have much of an effect on the improvement in the transverse properties of the composite, which is mainly determined by the matrix phase.

**Figure 16:**
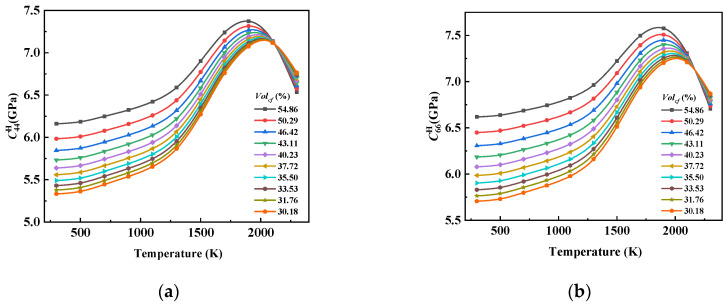
The influence curve of temperature change on (**a**) $C_{44}^{H}$ and (**b**) $C_{66}^{H}$.

Figure 16 shows that the shear modulus of the composite material under temperature load presented a similar change rule to the elastic coefficients of graphite. Based on the theory of micromechanics [46], the shear properties of composite materials are mainly affected by the matrix, and the fiber properties have no obvious effect on the shear properties of composite materials under the conventional fiber bulk ratio.

#### 5.1.2. CTE

**Figure 17:**
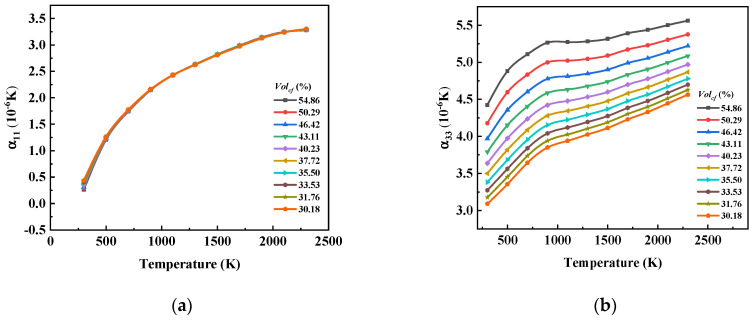
The influence curve of temperature change on (**a**) $\alpha_{11}$ and (**b**) $\alpha_{33}$.

Figure 17 shows that both $\alpha_{11}$ and $\alpha_{33}$ increased with the increase in temperature, and the change curves showed obvious nonlinear characteristics. The increasing trend of $\alpha_{11}$ with increasing temperature tended to be gentle, while the increasing trend of $\alpha_{33}$ with increasing temperature had obvious stage characteristics. The growth rate of $\alpha_{33}$ before 1000 K was obviously higher than that after 1000 K. In addition, $Vol_{cf}$ had little effect on $\alpha_{11}$, but $\alpha_{33}$ was greatly affected by $Vol_{cf}$.

**Figure 18:**
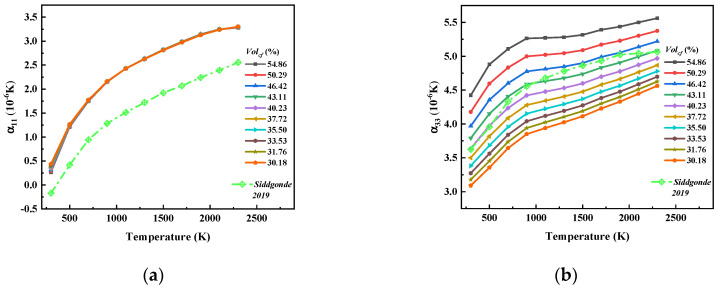
Comparison CTE of 8HSC/Cs between this paper and Ref. [32]. (**a**,**b**) Where, (**a**) $\alpha_{11}$ and (**b**) $\alpha_{33}$.

Figure 18 shows a comparison between the CTE of the 8HS C/C composites calculated using the asymptotic homogenization method in this paper and the CTE of the 5HS C/C composites calculated using the FEM method in Ref. [32]. Due to the different volume fractions of the carbon fiber bundles used in this paper and in Ref. [32] (74.2% in this paper and 80% in Ref. [32]) as well as the different performances of the carbon fiber bundles and the different volume fractions of the carbon fibers woven into the C/C composites (not provided in Ref. [32]), the weaving methods were also different (8HS in this paper and 5HS in Ref. [32]). Therefore, it was not possible to quantitatively evaluate the accuracy of the calculation results in this paper based on the literature results, but we can consider that the calculation results in present work conformed to the trend of CTE of 2D woven C/C composites changing with temperature.

### 5.2. Effect of Volume Fraction of Carbon Fiber on Thermo-Mechanical Properties of Composites

#### 5.2.1. Elastic Coefficient

To discuss the effect of $Vol_{cf}$ on the material properties, ten different models of $Vol_{cf}$ varying from 30% to 55% were established, and the increase in $Vol_{cf}$ was not uniform. The following is the law of the material-related properties changing with $Vol_{cf}$ under different temperature conditions.

With the increase in $Vol_{cf}$, the elastic properties of the 8HS C/C composites under different temperature conditions showed nearly linear changes, but the changes were not consistent.

**Figure 19:**
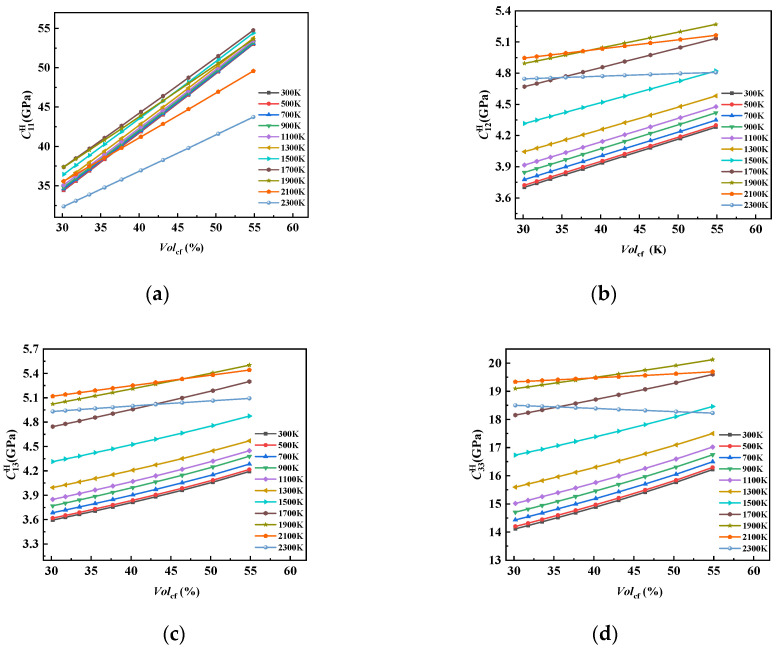
The influence curve of changes in $Vol_{cf}$ on (**a**) $C_{11}^{H}$, (**b**) $C_{12}^{H}$, (**c**) $C_{13}^{H}$, and (**d**) $C_{33}^{H}$.

Among them, Figure 19 shows that $C_{11}^{H}$ increased nearly linearly with the increase in $Vol_{cf}$ at different temperatures, but the performance growth trend was different at different temperatures. In the range of 300–1700 K, the performance increased nearly linearly with the increase in $Vol_{cf}$, and the curve slope values were similar. $C_{11}^{H}$ was mainly affected by the properties of the carbon fiber, and the axial elastic coefficients of the carbon fiber were much larger than those of graphite, resulting in an increase in $C_{11}^{H}$ with the increase in $Vol_{cf}$ at different temperatures. However, after 1700 K, due to the decline in the carbon fiber properties, the growth trend of $C_{11}^{H}$ with $Vol_{cf}$ slowed down. In addition, $C_{11}^{H}$ at the temperature of 1700 K and 1900 K was close in value, but due to the difference in the slope of the near-linear curve, the two curves intersected with the increase in the fiber volume fraction, and the intersection point appeared at about $Vol_{cf}=30\%$, after which the value peak appeared at the temperature point of 1700 K.

Different from $C_{11}^{H}$, which as mainly affected by the performance of the carbon fiber, $C_{12}^{H}$, $C_{13}^{H}$, and $C_{33}^{H}$ mainly depended on the performance of the graphite matrix, which resulted in their values and growth rates being much smaller than those of $C_{11}^{H}$. At the same time, due to the difference in the performance of the reinforced phase and the matrix phase at the ultra-high temperature, the growth rates of $C_{12}^{H}$, $C_{13}^{H}$, and $C_{33}^{H}$ slowed down around 1700 K, showing a downward trend. As a result, at 2300 K, $C_{12}^{H}$ and $C_{13}^{H}$ were almost unaffected by $Vol_{cf}$, while $C_{33}^{H}$ decreased with the increase in $Vol_{cf}$.

**Figure 20:**
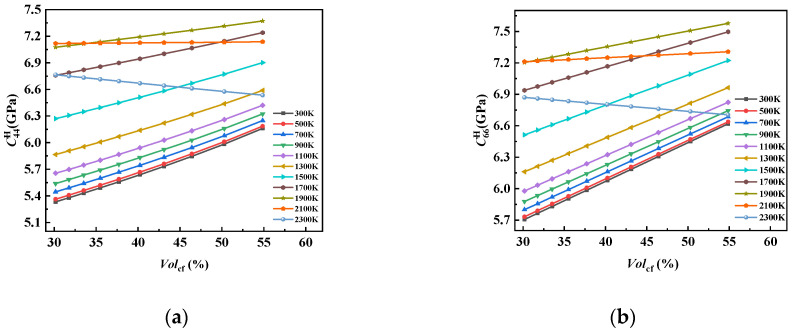
The influence curve of $Vol_{cf}$ change on (**a**) $C_{44}^{H}$ and (**b**) $C_{66}^{H}$.

The influence curves of $Vol_{cf}$ on $C_{44}^{H}$ and $C_{66}^{H}$ at different temperatures (Figure 20) were similar to those of $C_{33}^{H}$, but there were differences in the values and individual temperatures. It can be seen from the slope of the near-linear curve that compared with $C_{33}^{H}$, the trend of $C_{44}^{H}$ decreasing with the increase in $Vol_{cf}$ at 2300 K was slowed down. This was due to the fact that $C_{44}^{H}$, which is related to the shear properties of the composite, was more affected by the matrix graphite, and although the performance of the carbon fiber bundle decreased sharply by more than 30% in the temperature range from 300 K to 2300 K, the value of $C_{44}^{H}$ only decreased by about 3.4%, and the same $C_{66}^{H}$ only decreased by about 2.4%.

#### 5.2.2. CTE

**Figure 21:**
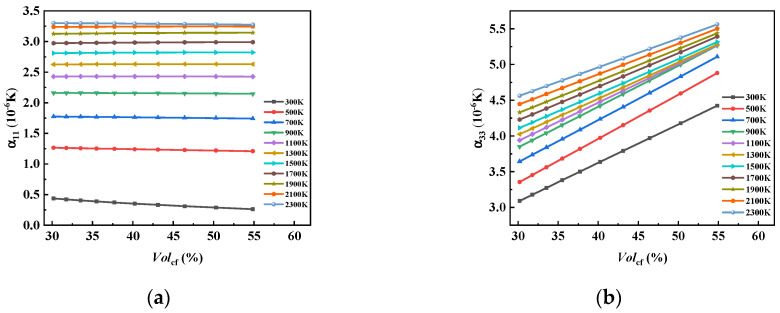
The influence curve of changes in $Vol_{cf}$ on (**a**) $\alpha_{11}$ and (**b**) $\alpha_{33}$.

From Figure 21, we can see that the change in $Vol_{cf}$ had no obvious effect on $\alpha_{11}$. With the increase in temperature, the influence curve tended to be more horizontal. $\alpha_{11}$ was most significantly affected by $Vol_{cf}$ at a temperature of 300 K, and it decreased with the increase in $Vol_{cf}$ from 0.438 × 10^−6^ to 0.261 × 10^−6^. It is predicted that $\alpha_{11}$ will approach zero with the continuous increase in $Vol_{cf}$. $\alpha_{33}$ increased significantly with the increase in $Vol_{cf}$, and the impact of changes in $Vol_{cf}$ at different temperatures was different.

### 5.3. Effect of Weaving Mode on Elastic Properties of Composites

In this section, we discuss the effect of different satin weaving methods on the elastic coefficient of the C/C composites by establishing an RVE with the same fiber bundle size and containing the same fiber volume fraction (35.5%, 46.42%) for the different weaving methods (4HS and 8HS).

**Figure 22:**
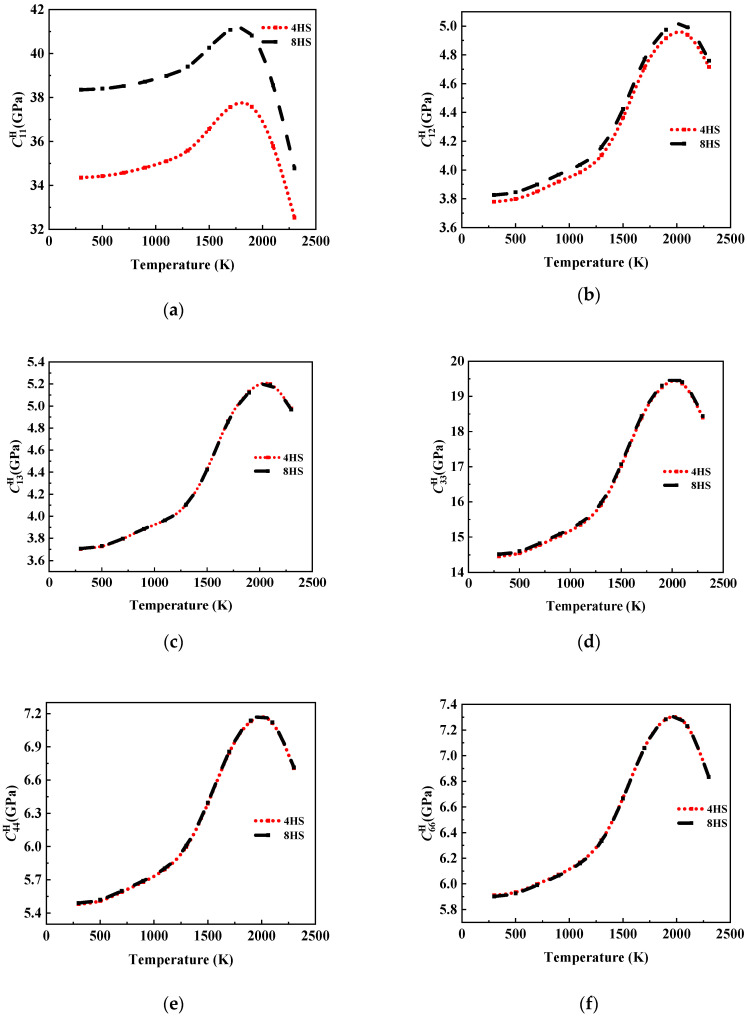
Elastic properties of 4HS C/C composites and 8HS C/C composites. The composites were affected by different temperatures, with $Vol_{cf}=30.50\%$. (**a**–**f**) Where, (**a**) $C_{11}^{H}$, (**b**) $C_{12}^{H}$, (**c**) $C_{13}^{H}$, (**d**) $C_{33}^{H}$, (**e**) $C_{44}^{H}$, and (**f**) $C_{66}^{H}$.

**Figure 23:**
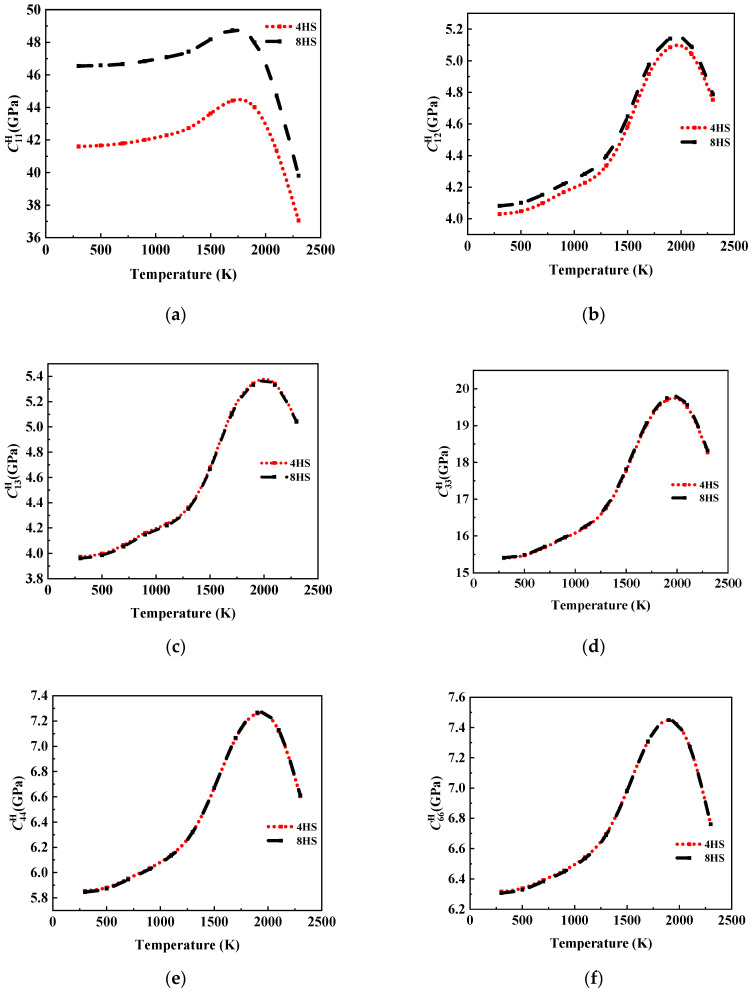
Elastic properties of 4HS C/C composites and 8HS C/C composites. The composites were affected by different temperatures, with $Vol_{cf}=46.42\%$. (**a**–**f**) Where, (**a**) $C_{11}^{H}$, (**b**) $C_{12}^{H}$, (**c**) $C_{13}^{H}$, (**d**) $C_{33}^{H}$, (**e**) $C_{44}^{H}$, and (**f**) $C_{66}^{H}$.

The temperature influence curves of the C/C composites corresponding to 4HS and 8HS are illustrated in Figure 22 and Figure 23. It can be seen that the values of $C_{11}^{H}$ and $C_{12}^{H}$ of the 4HS C/C composites were smaller than those of the 8HS C/C composites, and there was no significant difference in $C_{13}^{H}$ and $C_{33}^{H}$ between the two; similarly, there was no obvious change in $C_{44}^{H}$ and $C_{66}^{H}$ related to shear performance. It can be considered that the elastic properties of the 8HS C/C composites were slightly better than that of the 4HS C/C composites, which is consistent with the experiments in Xu’s work [33].

The analysis shows that the horizontal properties of the braided fiber composites were mainly affected by the fiber properties. For the 4HS C/C composites, the interweaving frequency of warp and zonal fiber bundles was larger, and the bending portion of the fiber bundles was higher. Therefore, the elastic properties of the composite in the X and Y directions were slightly lower than those of the 8HS C/C composites. However, the Z-direction properties of the composites mainly depended on the properties of the graphite matrix, and the fiber weaving mode had no obvious effect on it. Similarly, the shear properties of the composite also depended on the properties of the graphite matrix, and the carbon fiber phase only had a significant effect on the shear properties of the composite when the fiber proportion was extremely high. Therefore, when $Vol_{cf}$ was 35.5% and 46.2%, there was no significant difference in the values of $C_{44}^{H}$ and $C_{66}^{H}$ of the 4HS C/C composites and 8HS C/C composites.

## 6. Conclusions

In this paper, the microstructure of 8HS C/C composites was characterized, and the distribution and structure size of the carbon fiber phase were obtained. In addition, RVE models with different volume fractions of carbon fibers were constructed. The elasticity coefficient and the CTE of 8HS C/C composites at various temperatures were predicted using asymptotic homogenization and the finite element method. The effects of temperature and the material content on the properties of the 8HS C/C composites were discussed, and the dependence of the elastic constants and the CTE of the 8HS C/C composites on temperature was investigated. Furthermore, the properties of 4HS and 8HS composites with the same fiber content were studied. The main conclusions are as follows:

(1) The temperature change had a significant effect on the elasticity constants and the CTE of the composite. The rule of $C_{11}^{H}$ affected by temperature showed that there was no obvious change at a temperature of 500 K. With the increase in temperature, the value of $C_{11}^{H}$ first increased and then decreased under the influence of the carbon fiber and graphite, and it reached an extreme value at 1500 K. The other stiffness matrix elements were more obviously affected by the graphite matrix, and the temperature of the extreme value was different. $\alpha_{11}$ and $\alpha_{33}$ also increased with the increase in temperature, but the behavior of $\alpha_{11}$ with temperature presented obvious nonlinearity, while the value of $\alpha_{33}$ increased with temperature approximately in two linear stages with a boundary of 1000 K.
(2) The influence of $Vol_{cf}$ on the elastic constants and CTE was not similar, and $C_{11}^{H}$ showed a nearly linear increase with the increase in $Vol_{cf}$ in all the temperature ranges studied. The other elastic coefficients first increased and then decreased with the increase in temperature and $Vol_{cf}$. $\alpha_{11}$ was almost unaffected by changes in $Vol_{cf}$ except at a temperature of 300 K, and $\alpha_{33}$ exhibited a nearly linear increase with increasing $Vol_{cf}$.
(3) Regarding the influence of the weaving mode on the material properties, it was observed that the elastic properties of the 4HS C/C composites were slightly lower compared to those of the 8HS C/C composites due to the more frequent interweaving and a higher proportion of bent fiber regions, while the shear properties showed no significant difference.

## Figures and Tables

**Table 1:** Comparison between the calculation results in this paper and the experimental results of others.

	Present Work	Reference [40]	Relative Error
Longitude direction (X)	27.35	27.5	0.55%
Latitude direction (Y)	24.43	25.9	5.67%

**Table 2:** Fiber parameters.

Item	Data
Size of RVE L×W×H	9.6×9.6×0.72 mm^3^
Bundle width a	1.08 mm
Bundle thickness b	0.24 mm
Interfascicular distance d	1.2 mm

**Table 7:** Elastic coefficient matrix of the extended RVE model.

Number	*x*	*y*	*z*	*C*_11_	*C*_12_	*C*_13_	*C*_33_	*C*_44_	*C*_66_
I	0	0	0	49.482	4.172	4.062	15.767	6.449	5.984
II	1	0	0	49.483	4.174	4.067	15.788	6.449	5.988
III	2	0	0	49.484	4.174	4.069	15.795	6.449	5.989
IV	0	1	0	49.484	4.174	4.067	15.788	6.449	5.986
V	0	2	0	49.485	4.174	4.069	15.795	6.449	5.987
VI	0	0	1	49.486	4.175	4.073	15.807	6.449	5.990
VII	0	0	2	49.488	4.176	4.076	15.821	6.449	5.992
VIII	1	1	0	49.486	4.175	4.073	15.808	6.449	5.990
IX	1	0	1	49.487	4.176	4.076	15.818	6.449	5.992
X	0	1	1	49.488	4.176	4.075	15.818	6.449	5.991
XI	1	1	1	49.489	4.176	4.078	15.829	6.449	5.993
Average				49.486	4.175	4.072	15.807	6.449	5.990
Standard Error				0.00181	9.89 × 10^−4^	0.00394	0.01489	3.76 × 10^−5^	0.00232

**Table 8:** Model parameters for mesh sensitivity analysis.

Model Number	Number of Equidistant Scattered Points *S*	Mesh Size	X-Direction Fiber		Y-Direction Fiber		Carbon Matrix		Mesh Total
			Number of Mesh	Vol.%	Number of Mesh	Vol.%	Number of Mesh	Vol.%	
A	80	0.12	12,112	31.54	12,112	31.54	14,176	36.92	38,400
C	100	0.096	19,628	24.54	22,988	28.74	37,384	46.73	80,000
E	120	0.08	36,608	28.25	36,704	28.32	56,288	43.43	129,600
G	140	0.0686	49,324	25.17	52,276	26.67	94,400	48.16	196,000
I	160	0.06	77,232	25.14	77,264	25.15	152,704	49.71	307,200
J	170	0.0565	89,598	25.84	83,502	24.08	173,700	50.09	346,800
K	180	0.0533	109,684	26.04	104,972	24.92	206,544	49.04	421,200
L	200	0.048	153,104	25.52	153,136	25.52	293,760	48.96	600,000
M	240	0.04	260,000	25.08	260,000	25.08	516,800	49.85	1,036,800

## Data Availability

Data are contained within the article.
